# Transcriptome Analysis of Glutathione Response: RNA-Seq Provides Insights into Balance between Antioxidant Response and Glucosinolate Metabolism

**DOI:** 10.3390/antiox11071322

**Published:** 2022-07-05

**Authors:** Biao Zhu, Kuanhong Wang, Zhile Liang, Zhujun Zhu, Jing Yang

**Affiliations:** Key Laboratory of Quality and Safety Control for Subtropical Fruit and Vegetable, Ministry of Agriculture and Rural Affairs, Collaborative Innovation Center for Efficient and Green Production of Agriculture in Mountainous Areas of Zhejiang Province, College of Horticulture Science, Zhejiang A&F University, Hangzhou 311300, China; billzhu@zafu.edu.cn (B.Z.); 2019101032006@stu.zafu.edu.cn (K.W.); liangzl@stu.zafu.edu.cn (Z.L.)

**Keywords:** glutathione (GSH), antioxidant system, glucosinolate metabolism, pakchoi

## Abstract

When being stressed, plants require a balance between the resistance pathway and metabolism. Glucosinolates (GS) are secondary metabolics that widely exist in Brassicaceae. Glutathione (GSH) not only participates in plant processing reactive oxygen species (ROS) but also directly participates in GS synthesis as a sulfur donor. Therefore, we used transcriptomic to identify antioxidant and GS metabolism responses in GSH-treated pakchoi. Our study elucidated that GSH can be used as priming to improve oxidative resistance and preferentially stimulate the expression of resistance genes such as *CAT1*. The reduction in transcription factor expression inhibits the key steps of the GS synthesis pathway. When ROS returned to normal level, the resistance gene decreased and returned to normal level, while GSH restored the gene expression of GS biosynthesis. This work puts forward the mechanism of GSH in regulating the antioxidant system and glucosinolate metabolic pathway, which provides a basis for further study on the relationship between environmental signals and plant metabolism and provides ideas for follow-up research.

## 1. Introduction

Plants in their natural environment are continuously being threatened by a range of stress factors, including various abiotic stress conditions such as extreme temperatures, heavy metals, drought, and salinity, which can lead to the generation of ROS and alter the intracellular redox homeostasis [1,2]. To prevent the damage caused by excessive ROS under adverse conditions, plants have two kinds of ROS-free radical processing systems, enzymatic and non-enzymatic, which play an important role in protecting the structure and integrity of the membrane and resisting the damage of ROS-free radicals to the membrane [3,4]. Nonenzymatic antioxidants (ascorbate (AsA), glutathione (GSH), α-tocopherol, phenolic compounds, alkaloids, and non-protein amino acids) and antioxidant enzymes ((superoxide dismutase (SOD), catalase (CAT), ascorbate peroxidase (APX), monodehydroascorbate reductase (MDHAR), dehydroascorbate reductase (DHAR), glutathione reductase (GR), glutathione peroxidase (GPX), glutathione S-transferase (GST), and peroxidases (POD)) function to scavenge ROS and decrease their levels under stress conditions [3,5,6,7,8,9,10,11,12,13].

GSH is one of the non-protein tripeptide thiol compounds [14]. Known as the ‘master antioxidant’ or ‘super defender’, GSH is an important antioxidant substance, which is directly or indirectly involved in the detoxification of ROS in plants [15,16,17]. During this process, GSH is oxidized to glutathione disulfide (GSSG), thus restoring the high ratio of GSH/GSSG caused by ROS. GSH not only participates in the direct detoxification of ROS but also plays an indirect role by regulating the activities of antioxidant enzyme systems, such as GPX and GST [7,18,19]. In addition, GSH participates in redox signal transduction and protects cells from stress by activating phenolic compounds and terpenes such as carotenoids [20,21,22,23]. To construct different redox levels, GSH, GSSG, glutathione inhibitor (BSO), and strong reductant (DTT) were sprayed on pakchoi in this study.

Notably, various specialized plant secondary metabolites (e.g., phenolic compounds and carotenoids) are produced in response to diverse external stimuli and changing environmental conditions [24]. Although many studies focus on the relationship between abiotic stress and plant growth, the current knowledge regarding interactions between stress response and plant secondary metabolites is relatively preliminary and fragmented [25].

Glucosinolates (GS) have attained importance in recent years as a new class of secondary metabolites of profound physiological properties. Mainly found in the Brassicaceae plants, this nitrogen- and sulfur-containing secondary metabolite plays an important role in the plant immune system, human health, and so on [26,27,28]. The relationship between GSH and GS synthesis mainly comes from two aspects. Firstly, GSH can provide a reducing sulfur donor for GS synthesis; secondly, GSH can indirectly affect the synthesis of GS by regulating the primary sulfur metabolism [29,30].Our previous study found that sulfur metabolism and GS synthesis showed opposite trends under GSH and GSSG treatment, which might be because plants under GSSG treatment were induced by redox signal and promoted GS synthesis.

Pakchoi (*Brassica* *chinensis* L.) is one of the most popular and important leafy vegetables because of its high nutritional value, rapid growth, and low production cost across Asia [31,32].In this study, we adopted RNA sequencing (RNA-Seq) to identify GSH response in pakchoi. Transcriptomic, gene expression profile, and enzyme activity showed that GSH has a broad-spectrum resistance, especially in the antioxidant system. Meanwhile, the secondary metabolic pathway and the pathway related to plant growth and development are inhibited, especially the GS metabolic pathway. In addition, the jasmonic acid (JA) synthesis pathway and resistance-related genes are inhibited in the early stage. Therefore, by analyzing transcription factors, we speculate that GSH may inhibit GS synthesis by regulating the JA signal pathway, to regulate the balance between the antioxidant system and secondary metabolism in plants.

## 2. Materials and Methods

### 2.1. Growth Condition and Treatments

The seeds of pakchoi (*Brassica rapa* L. ssp. *chinensis* var. *communis*. cv. Hangzhou You Dong Er) were used in this study. All plants were germinated in vermiculite for two days. Then, the seedlings were transferred to a growth chamber and, when they reached the six-leaf stage, were transferred to 8 cm plastic pots filled with cultivation medium (compost: peat: sand (1:1:1, *v*/*v*/*v*)). The plants were grown with a temperature of 20–25 °C, a relative humidity (RH) of 60% (day) and 80% (night), a 16 h light and 8 h dark cycle. Each plastic pot in which unbolted plants were growing was put into a plastic bag for one day to adapt the plant to the sealed environment.

After the acclimation, a water emulsion of each 100 μM GSH (Sigma-Aldrich, Saint Louis, MO, USA; Cat Nb G4251-1G), 100 μM GSSG (Sigma-Aldrich, Saint Louis, MO, USA; Cat Nb G4376-500MG), 100 mM BSO (Sigma-Aldrich, Saint Louis, MO, USA; Cat Nb B2640-500MG) and 5 mM DTT (Sigma-Aldrich, Saint Louis, MO, USA; Cat Nb D0632-1G) was sprayed on the plants with a hand-pump aerosol spray bottle (500 mL per pot)at 7:00 a.m., then incubated for 1 h, 3 h, 6 h, 12 h, and 24 h to transcriptomic analysis, 6 h, 12 h and 24 h to physiological analysis. The harvested tissue was stored at −80 °C until further processing.

### 2.2. Measurement of Lipid Peroxidation

The level of lipid peroxidation was measured by estimating malondialdehyde (MDA), a decomposition product of the per-oxidized polyunsaturated fatty acid composition of the membrane lipid, using 2-thiobarbituric acid (TBA) as the reactive material following the method of Dhindsa [33]. The tissue was homogenized with ice-cold PBS and then was centrifuged at 6000× *g* at 4 °C. The supernatant was mixed with a reaction mixture of thiobarbituric acid (TBA) and TCA and heated in a water bath. Then the cooled supernatant mixture was read at 450 nm, 532 nm, and 600 nm. The concentration of MDA was calculated by using the extinction coefficient of 155 mM^−1^cm^−1^ and was expressed as μmol of MDA L^−1^.

### 2.3. Glutathione Content Assay

GSH is a typical sulfhydryl compound. Dithiocarbenzoic acid can react with sulfhydryl compounds to produce yellow compounds. The contents of GSH and GSSG can be determined by colorimetry. According to this principle, GSH and GSSG content of pakchoi leaves were measured using the GSH and GSSG determination kit (Nanjing Jiancheng Bioengineering Institute, Nanjing, China) following the manufacturer’s instructions.

### 2.4. Enzymatic Antioxidant Systems

The activities of superoxide dismutase (SOD), peroxidase (POD), catalase (CAT), ascorbate peroxidase (APX), dehydroascorbate reductase (DHAR), and glutathione reductase (GR) in leaves were analyzed using the following methods.

Superoxide dismutase (SOD; EC 1.15.1.1) activity was determined using the method of Giannopolitis with minor modifications [34]. Approximately 0.30 g fresh pakchoi leaves were ground on ice in 3 mL extraction buffer containing 50 mM sodium phosphate (pH 7.8), and the homogenate was centrifuged at 6000× *g* for 10 min at 4 °C. A reaction mixture containing 1.5 mL 50 mM sodium phosphate (pH 7.8), 300 mL 130 mM methionine, 300 mL 750 mM nitro-blue tetrazolium (NBT), 300 mL 100 mM EDTA-Na2, 300 mL 20 mM riboflavin, 100 mL enzyme extract, and 100 mL distilled water was illuminated for 20 min and the sample absorbance was determined at 560 nm. One unit of SOD activity was defined as the amount of enzyme corresponding to 50% inhibition of the NBT reduction.

The method of determining peroxidase (POD; EC 1.11.1.7) activity was as described by Hemeda with minor modifications [35]. Approximately 0.30 g fresh pakchoi leaves were ground on ice in 5 mL ice-cold extraction buffer containing 50 mM sodium phosphate (pH 5.5), and the homogenate was centrifuged at 6000× *g* for 10 min at 4 °C. The reaction mixture containing 1.0 mL 50 mM sodium phosphate (pH 5.5), 1.0 mL 0.3% H_2_O_2_, 0.95 mL 0.2% guaiacol, 50 mL enzyme extract. The absorbance value of 470 nm changes in 0.01 per second as a unit of POD enzyme activity.

Catalase (CAT; EC 1.11.1.6) activity assay followed the method of Hasanuzzaman et al. [36]. Approximately 0.30 g of fresh pakchoi leaves were ground on ice in a 5 mL extraction buffer containing 50 mM sodium phosphate (pH 7.8), and the homogenate was centrifuged at 6000× *g* for 10 min at 4 °C. The reaction mixture containing 1.5 mL 25 mM sodium phosphate (pH 7.8), 300 mL 0.1 M H_2_O_2_, 200 mL enzyme extract and 1.0 mL distilled water. The absorbance value of 240 nm changes in 0.1 per minute as a unit of CAT enzyme activity.

To assess ascorbate peroxidase (APX; EC 1.11.1.11) activity, approximately 0.30 g fresh pakchoi leaves were extracted by 5.0 mL cold 50 mM sodium phosphate (pH 5.5) containing 0.2 mM EDTA-Na2, then the homogenate was centrifuged at 6000× *g* for 10 min at 4 °C. The supernatant was used to determine APX activity using the method of Nakano [37].

Dehydroascorbate reductase (DHAR; EC: 1.8.5.1) activity was assayed according to Nakano and Asada by using dehydroascorbate (DHA), phosphatebuffer (pH 7.0), GSH, and EDTA; absorbance was recorded at 265 nm and the extinction coefficient 14 mM^−1^cm^−1^ was considered for calculating DHAR activity [37].

The method of Foyer and Halliwell was used for glutathione reductase (GR, EC; 1.6.4.2) estimation, and glutathione-dependent oxidation of NADPH was monitored at 340 nm [38].

### 2.5. Estimation of Reactive Oxygen Species (ROS)

Hydrogen peroxide (H_2_O_2_) production in tissues was detected using DAB (3,3′-diaminobenzidine) (SolelyBio, 868272-85-9). Leaves in pakchoi were incubated in darkness for 24 h. They were then boiled in 95% ethanol to eliminate chlorophyll, and stored in fresh 95% ethanol, and imaged with a camera.

Superoxide(O_2_^•^^−^) production in tissues was detected using NBT (Nitro tetrazolium blue chloride) (SolelyBio, S19048-1g). Leaves in pakchoi were incubated in darkness for 6 h. Then, they were boiled in 95% ethanol to eliminate chlorophyll, and stored in fresh 95% ethanol, and imaged with a camera.

Molybdic acid reacts with H_2_O_2_ to form a complex whose amount can be measured at 405 nm, to calculate the H_2_O_2_ content. According to this principle, H_2_O_2_ content of pakchoi leaves was measured using the H_2_O_2_ determination Kit (Nanjing Jiancheng Bioengineering Institute, Nanjing, China) following the manufacturer’s instructions.

### 2.6. RNA Isolation, cDNA Library Preparation, Transcriptome Sequencing, RNA Sequencing Data Analysis, and Quantitative Real-Time PCR Analysis

After library preparation for transcriptome sequencing, using fastp v0.19.3 to filter the original data, mainly to remove reads with adapters. Clean reads were mapped to the pakchoi reference genome (PRJNA576336) using HISAT v2.1.0, and StringTie v1.3.4d was used for new gene prediction. Featurecounts v1.6.2 was used to calculate the gene comparison, and then calculate the FPKM of each gene according to the gene length. FPKM is the most commonly used method to estimate gene expression level. Differential expression analysis between the two groups was performed using DESeq2 v1.22.1, and *p* values were corrected using the Benjamin and Hochberg method. The corrected *p* value (FDR < 0.05) and |log2foldchange|(|FPKM| ≥ 1) are used as the threshold for significant difference expression. The enrichment analysis was performed based on the hypergeometric test. For KEGG, the hypergeometric distribution test was performed with the unit of pathway; for GO, it was performed based on the GO term. To validate the accuracy of the RNA-seq data, qRT-PCR analyses were performed on qTOWER3G fluorescence quantitative PCR instrument (qTOWER3G, Jena, Germany) using TB GREENTM Primix ex Taq TM II (TLI RNaseH plus) kit was used on qTOWER3G. The PCR amplification program was: 95 °C for 30 s (pre denaturation); 95 °C for 5 s, 60 °C for 30 s (40 cycles); 95 °C 5 s, 60 °C 30 s (MeltCurve). Fold changes of the gene expression level were calculated using the 2^−ΔΔCT^ method. The actin gene of pakchoi was used as a candidate reference gene. The multiple change of gene expression level was calculated by the CT method. The primers used in this study are shown in the Supplementary Table S1.

### 2.7. Measurement of Glucosinolates

The GS content was determined as previously described [39]. Briefly, 0.25 g lyophilized leaf powder was placed in a 10 mL glass tube to which 4 mL of 70% boiling methanol (75 °C) containing 200 μL of 5 mM sinigrin (Sigma-Aldrich Co., St. Louis, MO, USA) as an internal standard was added, and the mixture was placed in a water bath for 10 min. The solution was added with 1 mL of 0.4 mM barium acetate and then centrifuged at 8000 rpm for 10 min. The supernatant was transferred to a new centrifuge tube and the residue was extracted twice with 3 mL of 70% boiling methanol. Then, 5 mL extracts were loaded onto a 1 mL mini-column (JT Baker, Phillipsburg, NJ, USA) containing 0.5 mL of activated DEAE Sephadex™ A-25 (Amersham Biosciences, Uppsala, Sweden) and 2 mg mL^−1^ sulfatase (Sigma-Aldrich Co.). The resultant desulfoglucosinolates were eluted with 5 mL ultra-pure water. The eluants were passed through a 0.45 μm syringe filter and stored at −20 °C prior to analysis by HPLC. The GS profile was analyzed using an Agilent 1200 HPLC system (Agilent Technologies, Inc., Santa Clara, CA, USA) with a prontosil ODS2 column (250 × 4 mm, 5 μm, Bischoff, Leonberg, Germany). An aliquot (20 μL) of eluant was monitored with adiode array detector (G1315B) set at 229 nm at 30 °C. The mobile phase was ultra-pure water (A) and acetonitrile (Tedia, Fairfield, OH, USA) (B) in a linear gradient from 0 to 20% B in 34 min, then constant 20% B for 6 min, following by 100% B and 0% B prior to the injection of the next sample.

### 2.8. Statistical Analysis

Analysis of variance (ANOVA) procedures was carried out using SPSS software. The means were compared by Duncan when the difference was significant (*p* ≤ 0.05).In terms of RNA-seq data, DESeq2 v1.22.1 was used to analyze the differential expression between the two groups, and the *p* value was corrected using the Benjamini and Hochberg method. The corrected *p* value and |log2foldchange| are used as the threshold for significant difference expression.

## 3. Results

### 3.1. Identification of Pakchoi Transcripts in Response to GSH

**Figure 1:**
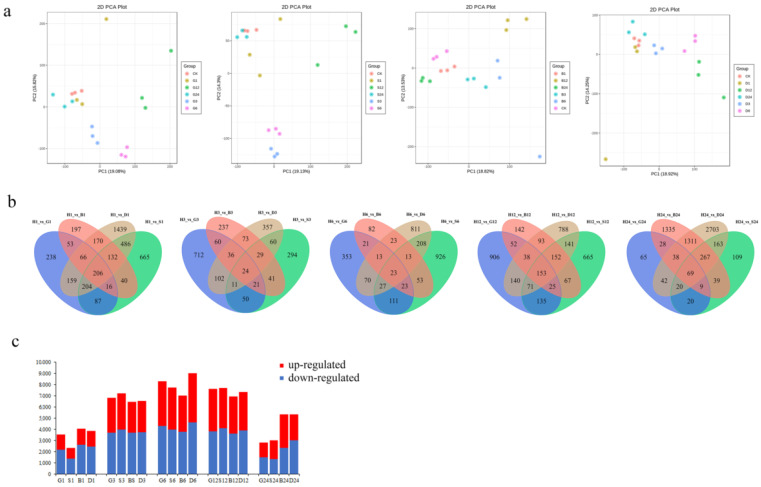
Transcriptomes of pakchoi under GSH treatments. (**a**) Principal component analysis (PCA) plots of transcripts identified by RNA-seq of pakchoi leaves treated with GSH, GSSG, BSO, and DTT at 1, 3, 6, 12, and 24 h. (**b**) Venn diagram illustrating the number of DEGs by GSH, GSSG, BSO, and DTT feeding over the time course. (**c**) Statistical table for the number of differential genes in different groups. Blue (down-regulated) and red (up-regulated) bars indicate differential genes (*p* < 0.01).

To identified genes that were differentially expressed under different spraying treatments and performed a genome-wide analysis of transcripts using high-throughput RNA-Seq technology. Transcript abundance was assessed in GSH-treated, GSSG-treated, BSO-treated, DTT-treated, and H_2_O-treated pakchoi leaves by microarray at 1 h, 3 h, 6 h, 12 h, and 24 h. Analysis at these five time points shows that the extent of the transcriptional response to the GSH, GSSG, BSO, and DTT treatments act significantly different. The gene expression level of each repeat was evaluated using principal component analysis (PCA) (Figure 1a). The samples from 1 h, 3 h, 6 h, 12 h, and 24 h after treatments clustered away from the 0 h (CK) samples, indicating that GSH, GSSG, BSO, and DTT spraying treatment induced changes in gene expression. All samples were detected and screened according to *p* < 0.05 and |log2foldchange| ≥ 1. A total of 24,591 differentially expressed genes (DEGs) were obtained for further analysis (Figure 1c). The number of up-regulated and down regulated genes at different time points under different treatments in this study is shown in Supplementary Table S2. Compared with untreated 0 h, the up-regulated genes at 1 h, 3 h, 6 h, 12 h, and 24 h of GSH spraying accounted for 62.10%, 54.15%, 52.15%, 50.33%, and 54.20% of the total DEG, respectively. The up-regulated genes at 1 h, 3 h, 6 h, 12 h, and 24 h after spraying GSSG accounted for 60.14%, 54.93%, 51.55%, 53.44%, and 44.69% of the total DEG, respectively. The up-regulated genes at 1 h, 3 h, 6 h, 12 h, and 24 h after spraying BSO accounted for 64.88%, 57.59%, 53.66%, 52.18%, and 43.74% of the total DEG, respectively. The up-regulated genes at 1 h, 3 h, 6 h, 12 h, and 24 h after spraying DTT accounted for 63.74%, 57.33%, 51.23%, 52.89%, and 56.74% of the total DEG, respectively. This showed that spraying treatment induced the expression of most GSH response genes in the early stage, which was helpful for pakchoi to respond quickly to stress.

### 3.2. GSH Increased the Oxidative Resistance of Pakchoi

**Figure 2:**
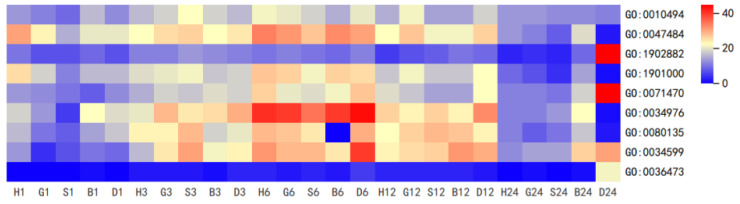
GO enrichment analysis of stress response to spraying treatments at 1, 3, 6, 12, and 24 h. Heat map represents the DEGs numbers (H, H_2_O treatment; G, GSH treatment; S, GSSG treatment; B, BSO treatment; D, DTT treatment).

DEGs were further analyzed by gene ontology (GO).By applying a hypergeometric test, GO term was found to be significantly enriched in DEGs compared with the whole genome background. Here, we found that all spraying treatments can produce broad-spectrum resistance (including ‘GO:0047484 regulation of response to osmotic stress’, ‘GO:1901000 regulation of response to salt stress’, ‘GO:0071470 cellular response to osmotic stress’, ‘GO:0034976 response to endoplasmic reticulum stress’, ‘GO:0080135 regulation of cellular response to stress’, and ‘GO:0034599 cellular response to oxidative stress’) to plants at 1–12 h, and reach the peak at 6 h after treatments. Interestingly, we found that DTT spraying still had a large number of gene expressed at 24 h, mainly focused on ‘GO:1902882 regulation of response to oxidative stress’, ‘GO:0071470 cellular response to osmotic stress’, ‘GO:0034599 cellular response to oxidative stress’, and ‘GO:0036473 cell death in response to oxidative stress’, while at this time, genes in GSH and GSSG treatment have basically returned to the pre-treatment level. This indicates that GSH can improve the broad-spectrum stress resistance of plants, especially oxidative stress resistance, while avoiding irreversible damage to plants (Figure 2).

**Figure 3:**
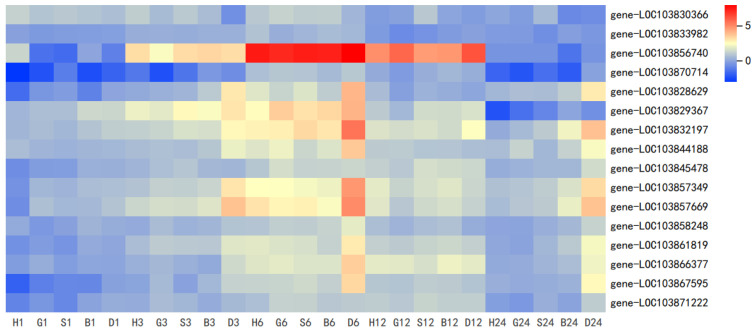
DEGs in stress response to spraying treatments at 1, 3, 6, 12, and 24 h. Heat map represents the value of DEGs as L2FC relative to CK (0 h) (H, H_2_O treatment; G, GSH treatment; S, GSSG treatment; B, BSO treatment; D, DTT treatment).

To verify whether GSH is related to broad-spectrum stress, we checked the gene expression of DEGs in these GO pathways.The results showed a similar trend to GO enrichment analysis (Figure 3). Details of DEGs are shown in Supplementary Table S3.

### 3.3. Coexpression Analysis Reveals GSH Involved in GS Metabolism and Antioxidant System in Pakchoi

The spraying treatments of GSH, GSSG, BSO, and DTT were analyzed by Kmeans cluster analysis and cluster heat map analysis. In Kmeans cluster analysis, the results noted that there are strong associations between GSH metabolism, antioxidant system, sulfur metabolism, plant hormone signaling pathway, and GS metabolism. In sprayed GSH, BSO and DTT samples, DEG of the above pathways clustered in the class of 24 h expression; DEGs in sprayed GSSG samples clustered in the category of 3 h expression. This indicates that different treatments have different responses to GSH metabolism, the antioxidant system, sulfur metabolism, plant hormone signaling pathway, and GS metabolic pathway, and GSSG spraying can more effectively activate GS metabolic pathway than other treatments (Supplementary Figure S1).

**Figure 4:**
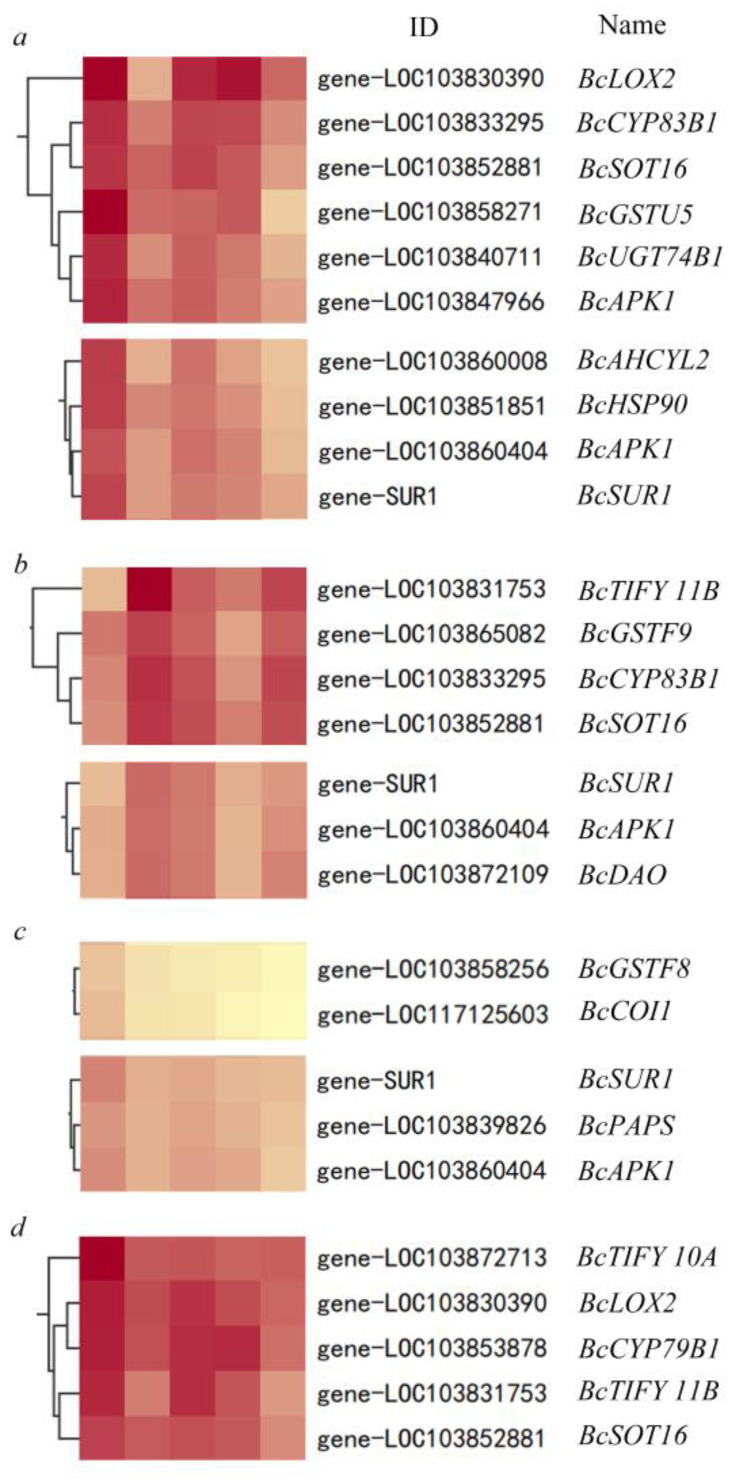
Enlarged view of the heat map analysis cluster of GSH (**a**), GSSG (**b**), BSO (**c**), and DTT (**d**) showing IDs and gene names.

By mapping the cluster heat map of different categories of genes clustered by different treatments of Kmeans, it was found that there was a high correlation between the GST family in GSH metabolism and adenylyl sulfate kinase (APK) family genes in sulfur metabolism pathway and GS-related gene expression. Compared with other treatments, *BcAPK1* was not strongly correlated with the GS metabolic pathway under DTT treatment, indicating that *BcAPK1* is the key gate for GSH to determine GS synthesis. GS will not be synthesized in large quantities without activating *BcAPK1* of sulfur metabolism. The heat map showed that GSH, GSSG, and BSO treatment activated the expression of *BcAPK1*, but the gene expression time of the GS metabolic pathway was different. Combined with the analysis of correlation and gene expression trend, we found that the expression of the GST family genes was strongly correlated with the expression of genes related to GS metabolism in time, that is, GSH directly affects GS synthesis through GSH, and then affects GS metabolism through sulfur metabolism, indicating that GST is the key point for GSH to determine the time of GS synthesis expression (Figure 4).

### 3.4. GSH Increased the Resistance of Oxidative Stress in Pakchoi

GSH can scavenge ROS in plants directly or indirectly.The above data also showed a strong linkage between GSH and antioxidant system. To demonstrate this, transcriptomic data, gene expression, enzyme activity, and markers of antioxidant system were examined.

#### 3.4.1. Effect of GSH on Oxidative Signaling in Pakchoi Leaves

**Figure 5:**
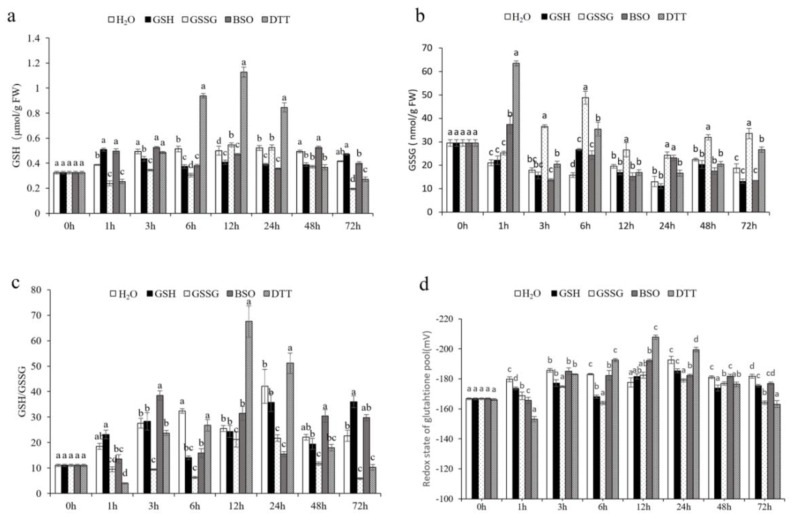
Content of GSH metabolism in leaves of pakchoi (*Brassicarapa* L. ssp. *chinensis*) plants treated with GSH (100 μM) or GSSG (100 μM) or BSO (1 mM) or DTT (5 mM). (**a**) Reduced glutathione (GSH). (**b**) Oxidized glutathione (GSSG). (**c**) Molar ratio of GSH/GSSG. (**d**) Redox state of the glutathione pool. Data are the means of three biological replicates (±SD) shown by vertical error bars. Different letters indicate significant differences according to Tukey’s test at 0.05% level.

The redox state of the GSH pool is a marker that indirectly shows the degree of oxidation in vivo. The higher the redox potential, the stronger the oxidation is, and the lower the redox potential, the reduction is mightier. The measurement showed that the increment of GSH redox potential was the most intense in the case of DTT treatment at 1 h. GSSG also presented strong antioxidant stress due to the GSH redox potential at 1, 3, 6, and 72 h after treatments. These suggested that GSSG and DTT can induce oxidative stress, in which DTT initiates stress before GSSG, but GSSG-induced stress is more persistent (Figure 5).

#### 3.4.2. Effect of GSH on Oxidative Stress Markers of Pakchoi Leaves

**Figure 6:**
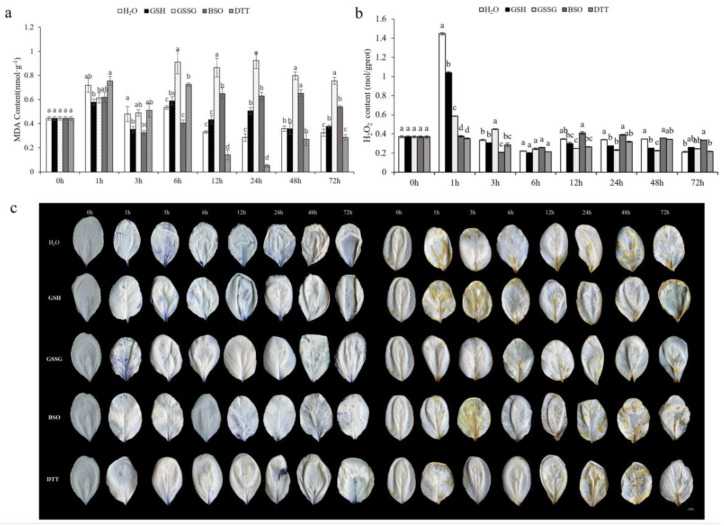
Effect of GSH on (**a**) lipid peroxidation (MDA), (**b**) hydrogen peroxide (H_2_O_2_), and (**c**) detection of ROS by Diaminobenzidine (DAB) and nitro-blue tetrazolium (NBT) staining under GSH, GSSG, BSO, and DTT treatment in pakchoi leaves. Data are the means of three biological replicates (±SD) shown by vertical error bars. Different letters indicate significant differences according to Tukey’s test at 0.05% level.

MDA is the end product of membrane lipid peroxidation, so the damage degree of cell membrane can be measured by its content. The MDA levels first increased 6 h after spraying GSH, GSSG, and BSO; 6 h after treatment with GSSG and DTT, the MDA level stretched significantly by 75% and 30%. Then, the MDA levels continued to rise, 12 h after GSSG and BSO sprinkling increased the MDA levels in pakchoi leaves by 171% and 96%, respectively, compared to H_2_O treatment, whereas the GSH treatment caused no significant change in MDA levels compared to H_2_O treatment at the same time. The highest peak of MDA content appeared 24 h after GSSG treatment, which progressed by 223% compared to H_2_O treatment (Figure 6a).

The hydrogen peroxide (H_2_O_2_) content was significantly increased in the treatments of H_2_O, GSH, GSSG, and BSO (Figure 6b). GSH and GSSG receivers displayed more DAB staining than did H_2_O receivers at 1 h after spraying. Compared with CK plants, the BSO receivers had a higher H_2_O_2_ content. This higher ROS accumulation in the GSH, GSSG, and BSO treatment was also confirmed by quantitative measurements of H_2_O_2_ (Figure 6c).

#### 3.4.3. Effect of GSH on Antioxidant Enzymes Activities of Pakchoi Leaves

**Figure 7:**
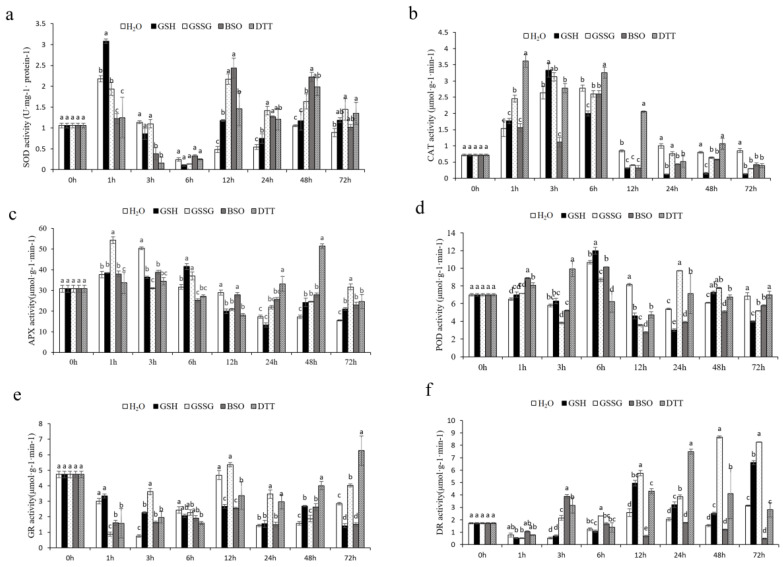
(**a**) Superoxide dismutase (SOD) activity, (**b**) catalase (CAT) activity, (**c**) ascorbate peroxidase (APX) activity, (**d**) peroxidase (POD) activity, (**e**) glutathione reductase (GR) activity, and (**f**) dehydroascorbate reductase (DHAR) activity in pakchoi leaves treated with GSH (100 μM) or GSSG (100 μM) or BSO (1 mM) or DTT(5 mM). Data are the means of three biological replicates (±SD) shown by vertical error bars. Different letters indicate significant differences according to Tukey’s test at 0.05% level. As part of the analysis of the potential interrelationship between GSH and antioxidant signaling, enzymatic components of the ascorbate–glutathione cycle were also studied. Figure 7 shows the activity of APX, DHAR, and GR. The performance of leaf APX activity increased 24 h after all treatments, especially at 48 h, DTT spraying uploaded the APX activity to 198%. GSSG-treated plants also showed an increase (103%) at 72 h. In response to GSSG, BSO, and DTT treatments, the leaf DHAR activity uptick at 3 h, except for GSH treatment. Treatment with GSH, GSSG, BSO, and DTT caused significant increases in enzyme activity of GR at 3 h, with GR level increasing by 197%, 376%, 114%,157% in the leaf respectively.

In general, antioxidant enzyme activities (SOD, POD, CAT, APX, GR, and DHAR) increased in response to the treatment of GSH (Figure 7). CAT and SOD are considered the first line of defense against an increase of ROS, especially in GSH treatment. Figure 5 shows the activity of both enzymes in leaves of pakchoi exposed to different conditions. In all assayed experimental conditions, an increase in both enzymatic activities in comparison to pakchoi was found. However, the maximum SOD activity increase was obtained in GSSG treatment at 24 h, the highest CAT activity was discovered in DTT treatment at 1 h.

The POD activity increased by 7%, 9%, 36%, and 23% in the leaf in response to spraying treatments, respectively, compared to the unstressed control in 1 h. The highest activity presented at 24 h in treatment with GSSG, increased the POD activity by 80% in the leaf above that observed in plants exposed to H_2_O. No significant changes were noted in leaf POD activity under GSH compared to the unstressed control.

#### 3.4.4. Effect of GSH on Antioxidant System Genes of Pakchoi Leaves

**Figure 8:**
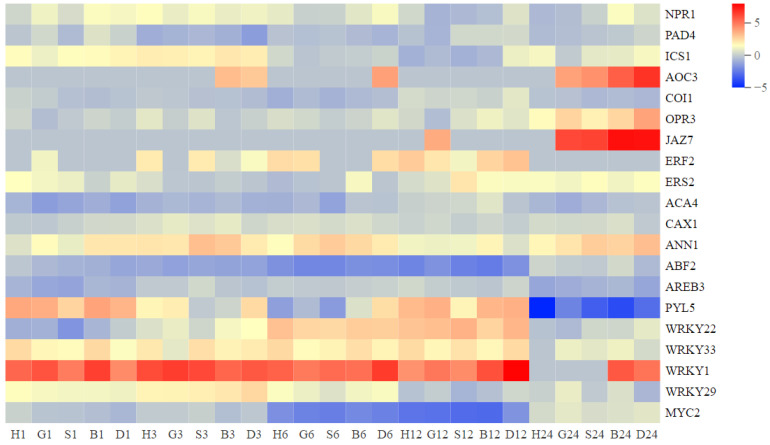
Typical resistance genes response to spraying treatments at 1, 3, 6, 12, and 24 h. Heat map represents the value of DEGs as L2FC relative to CK (0 h) (H, H_2_O treatment; G, GSH treatment; S, GSSG treatment; B, BSO treatment; D, DTT treatment).

Given the results above, we further investigated genes in the ROS processing system. Several terms associated specifically with oxidative stress were found, including ‘response to oxidative stress’, ‘hydrogen peroxide metabolic process’, ‘hydrogen peroxide biosynthetic process’, ‘reactive oxygen species biosynthetic process’, and ‘response to hydrogen peroxide’ in leaves (Figure 8). By searching the antioxidant system genes reported in the relevant literature and screening in combination with transcriptome data, values of DEGs in these GO terms are listed in Table 1, including common anti-oxidative genes such as *CAT1*, *GPX6*, *GR*, and *DHAR1*. This result revealed that the transcriptional response to GSH treatments in pakchoi comprises increased numbers of antioxidant-responsive genes relative to the control. Interestingly, 6–12 h after DTT treatment, the CSD family genes (*CSD1*) screened in DEG were not significantly expressed, while the expression under GSH, GSSG, and BSO treatment were significant, which is consistent with the activity of SOD. Therefore, CSD may be a key link in the pathway of the GSH-specific effect on the antioxidant system.

Typical resistance genes were also investigated, including the calcium signaling pathway and hormone signaling pathway (including SA, ET, and ABA) (Supplementary Figure S2). Combined with the expression of these resistance genes, we can roughly infer the path of GSH/GSSG affecting the antioxidant system. Firstly, in 1 h, spray treatment initiated RbohD-dependent ROS production PRX-related genes were highly expressed and activated the expression of *ANN1*, *PYL5*, *ERS2*, and *NPR1* in the calcium signal, ABA, ET, and SA signal pathways, respectively, indicating that GSH first activated the ROS receptor and affected the calcium signal, ABA, ET, and SA signal pathways. In the following 3 h, the related genes of CAT, GPX, GR, and APX in the ROS processing enzyme system were activated and reached the peak at 6 h. At this time, the resistance gene began to be highly expressed, indicating that the ROS processing enzyme system was activated and further activated the resistance system. The expression of SOD-related genes was concentrated in 12–24 h, and the genes of the JA pathway were also strongly expressed in 24 h, indicating that spraying treatment mainly caused H_2_O_2_ accumulation rather than O_2_^•−^. In addition, we also found that the performance of *NPR1* and *ANN1* in GSH, GSSG, and BSO treatments was different from DTT, suggesting that GSH may specifically affect the plant resistance system through the calcium and SA signal pathway.

### 3.5. GSH Inhibit the Secondary Metabolism during Oxidative Stress Response in Pakchoi

**Figure 9:**
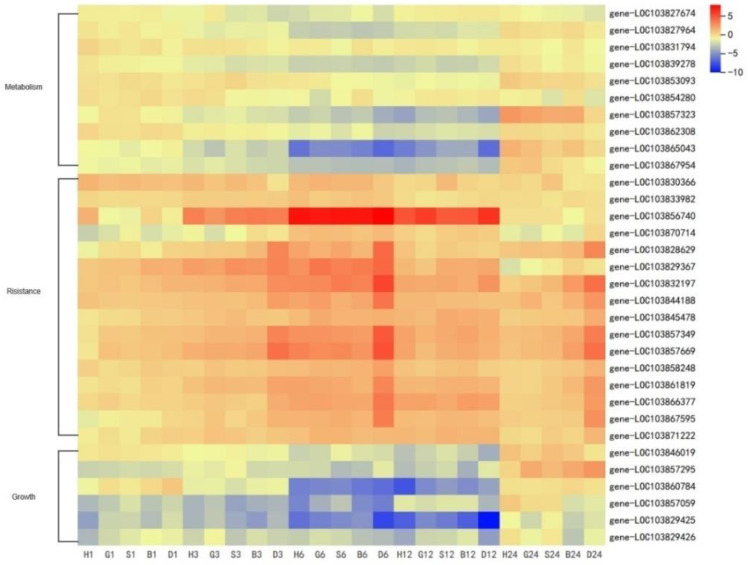
DEGs of metabolism, resistance, and growth response to spraying treatments at 1, 3, 6, 12, and 24 h. Heat map represents the value of DEGs as L2FC relative to CK (0 h) (H, H_2_O treatment; G, GSH treatment; S, GSSG treatment; B, BSO treatment; D, DTT treatment).

To investigate the relationship between antioxidant pathway and metabolism under stress, DEGs of ‘metabolism’ and ‘growth’ were selected (Figure 9, Table S4). The heat map intuitively shows that there is an antagonistic effect between antioxidant pathway and metabolic and growth pathway at 6–12 h. DEGs expressed in metabolism are mainly distributed in secondary metabolism, JA synthesis, tryptophan metabolism (GS synthesis), and anthocyanin biosynthesis. Growth-related DEGs are mainly distributed in SnRK2 in plant hormone signal transduction and *CYP73A* in cytochrome P450.That is, GSH can improve plant antioxidation while inhibiting the synthesis of secondary metabolites, especially GS. This provides an idea for us to find how GSH keeps the balance between metabolism and resistance pathway.

### 3.6. Effect of High Antioxidant Expression on GS Metabolic Pathway in Pakchoi

**Figure 10:**
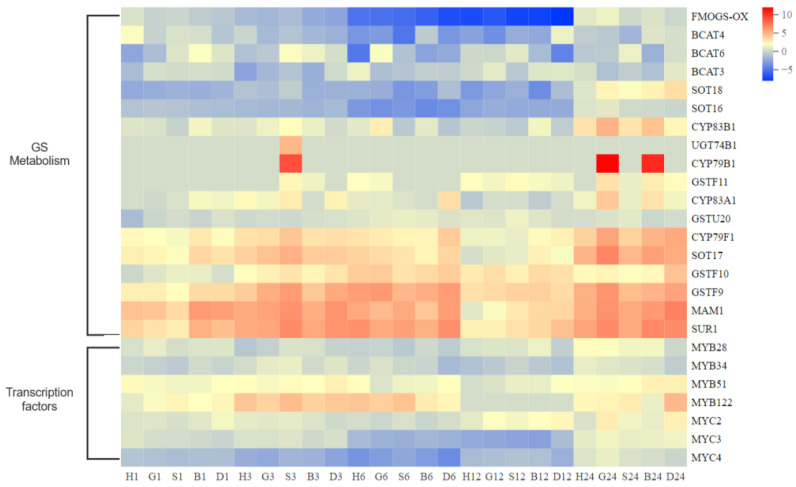
DEGs of metabolism, resistance, and growth response to spraying treatments at 1, 3, 6, 12, and 24 h. Heat map represents the value of DEGs as L2FC relative to CK (0 h) (H, H_2_O treatment; G, GSH treatment; S, GSSG treatment; B, BSO treatment; D, DTT treatment).

Due to the high response in treatments, we investigated the expression of genes related to GS metabolism (Figure 10). We observed that the genes down-regulated in 6–12 h were mainly BCAT and SOT family genes, which were related to the first and last steps of GS synthesis, respectively. In addition, we also detected that the gene expression of the first step of indole GS degradation was down-regulated. The up-regulated genes were concentrated in the middle of GS synthesis, and the expression decreased in 12 h. Most GS-related transcription factors were also inhibited in the early stage, and the expression was up-regulated in 24 h. Therefore, we speculate that GSH inhibits the key step of GS synthesis through transcription factors, so as to achieve the dynamic balance between antioxidant pathway and GS metabolic pathway.

**Figure 11:**
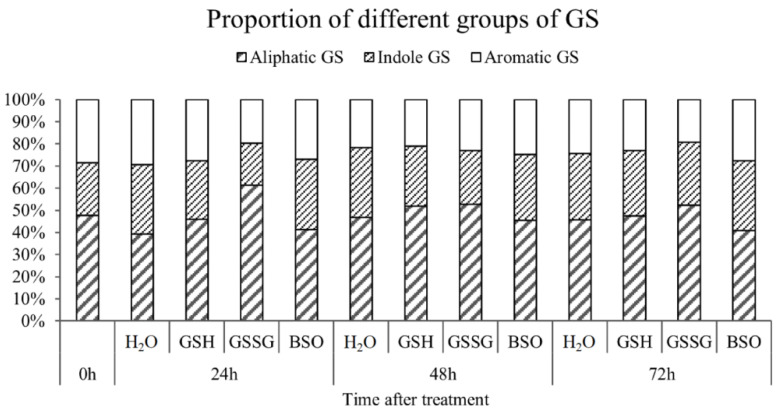
Effect of exogenous GSH/GSSG on the content of total GS and the proportion of different groups of GS in leaves of pakchoi.

We analyzed the changes in total GS, indole, aliphatic, and aromatic GS contents of pakchoi at 0, 24, 48, and 72 h after GSH, GSSG, and BSO treatment (as shown in Figure 11). After GSH treatment, the content of indole GS increased gradually, and the proportion of aliphatic GS first decreased at 24 h after treatment, and then began to increase at 48 h. Under GSH treatment, the content of total GS and indole GS showed the same change trend. Under the treatment of GSSG, the content of indole GS decreased significantly at 24 h and decreased by about 5% compared with CK. At 48 h, the content of indole GS increased and reached the highest at 72 h. The proportion of aliphatic GS increased significantly at 24 h after treatment, increased by 13% compared with CK, and then decreased at 48 h. The content of total GS increased gradually under GSSG treatment. Under BSO treatment, compared with CK, the proportion of aliphatic GS in total GS decreased, while the proportion of indole GS increased slightly at 24 h, and then maintained 32%. In conclusion, GSSG treatment can temporarily inhibit the content of indole GS, but the total GS content of pakchoi leaves is not significantly affected, indicating that the decrease in the GSH/GSSG ratio will specifically inhibit the synthesis of indole GS, which is consistent with the significant decrease of the expression of indole GS-related differentially expressed gene (*BcGSTF10*) at 6–12 h.

## 4. Discussion

When being stressed, a large number ofROS is generated [40]. ROS can cause peroxidation of lipids and oxidation of amino acids [41,42]. There are four common ROS: O_2_^•−^, H_2_O_2_, ·OH, and ^1^O_2_ [43]. GSH can participate in the processing of ROS and GS metabolism directly or indirectly [29,44,45]. GSH combines with metabolites to form a new metabolite to participate in the metabolic regulation of plants, which is the main mechanism of regulating many plant proteins. Many studies have revealed that GSH can directly participate in plant metabolism as a substrate [21,46,47].The relevant metabolism pathways include sulfur metabolism, protein synthesis, and NO storage. However, there are few studies on how GSH coordinates the expression of metabolism and resistance. In our study, the levels of secondary metabolism and antioxidant genes were gradually elevated with changing GSH/GSSG ratio in pakchoi. Moreover, we found that GSH can preferentially express resistance genes and inhibit metabolic pathways, especially secondary metabolic pathways.

Transcriptome data showed that GSH metabolism, the antioxidant system, and the GS metabolic pathway are related. According to correlation cluster analysis, GSH regulates GS metabolism through APK and GST family genes, and contributes differentially. APK family genes control GS synthesis, while GST family genes control the biosynthesis time of GS.

Combined with transcriptome analysis, we investigated how GSH keeps balance between the antioxidative system and GS metabolism in pakchoi. Six post-treatment timepoints (T = 0, 1, 3, 6, 12, and 24 h) were investigated, as the activities of pakchoi were different under five spraying conditions (H_2_O, GSH, GSSG, BSO, and DTT). Joined with KEGG and GO screening, we focused on the expression of genes, enzyme activity, and substance contents related to the antioxidant pathway and GS metabolic pathway. Exogenous GSH treatment of Young Loquat Fruits under low temperature stress can improve antioxidant capacity, inhibit chloroplast membrane lipid peroxidation, and improve overall cold tolerance [48]. Our results showed an early response to antioxidant pathway in GSH treatments, which verified the effect of exogenous GSH on increasing plant resistance by improving oxidative stress resistance in the present studies. In the regulation of gene expression, ROS receptor protein (*RbohD* and *Prx*) and SOD (*CSD1*) were the first to respond. After that, gene expression of most ROS processing enzymes was up−regulated. The detection of enzyme activities changed sensitively with the GSH/GSSG ratio, raised significantly when the GSH/GSSG ratio decreased, as with the level of oxidative stress markers. SOD is the first defense wall in oxidative damage in the cells and plays a key role in the alteration of O_2_^•−^ radicals to H_2_O_2_ and oxygen (O_2_) [49].Our study suggested that GSH may scavenge ROS through SOD (CSD) specifically. DTT is a particularly strong reducer because once in its oxidized state, it forms a very stablering structure with an internal disulfide bond, which makes it harder to oxidize back to its reduced state again [50].This is why we choose DTT as a comparison to GSH. Compared with the DTT treatment, we found it interesting that GSSG can activate many stress response systems (especially the ROS processing system) with the same degree of activation as DTT, while not causing irreversible damage to cells such as DTT. This indicates that GSH can improve the broad-spectrum stress resistance of plants while avoiding irreversible damage to plants, and may be used as priming to improve plant immunity.

A large number of secondary metabolism and resistant genes have been identified in our study, suggesting that GSH may participate in the coordination of metabolism and plant immune system. Therefore, we used gene expression profiles to analyze the relationship between these pathways. The results showed an obvious continuity between GS metabolism and antioxidant, that is, the expression of GS metabolic genes was inhibited when the expression of resistance genes was enhanced. This phenomenon appeared at 6–12 h after treatments. Subsequently, resistance gene expression was reduced and restored or lowered to original level. At this time, GS metabolic gene expression increased rapidly, especially the CYP79 and CYP83 family genes of cytochrome P450. In general, the series of reactions involved in GS biosynthesis can be further divided into three distinct phases, viz., chain elongation of the precursor amino acids, core GS structure formation, and side-chain modification reactions of the GS [51,52,53]. BCAT (*BCAT3*, *BCAT4*, *BCAT6*) and SOT (*SOT16*, *SOT18*) were significantly reduced in our study, which respectively responds to the first and last step of GS biosynthesis.Due to the early sampling time, only *FMO_GS-OX_* in GS degradation was identified, which also showed a significant decrease when the immune system is highly expressed. qPCR experiments further verified this finding.

Plant hormones such as jasmonic acid are major regulators of plant response to pathogen attack [54]. Previous studies showed that MeJA had a great inducing and long-lasting effect on GS accumulation [55,56]. When we classify and screen the gene expression of the antagonistic pathway, the JA signaling pathway seemed to be inhibited the same as the GS metabolism at early times after treatments. JA can inhibit the SOT family in GS synthesis through MYC transcripts [57,58].Results in our work verified the relationship between JA and GS synthesis on the one hand, and suggest that GSH may inhibit GS synthesis through the JA pathway, so as to achieve the balance between resistance and metabolism. Our work provides a useful basis for the development of studying the relationship between metabolism and resistance in plant stress response.

**Figure 12:**
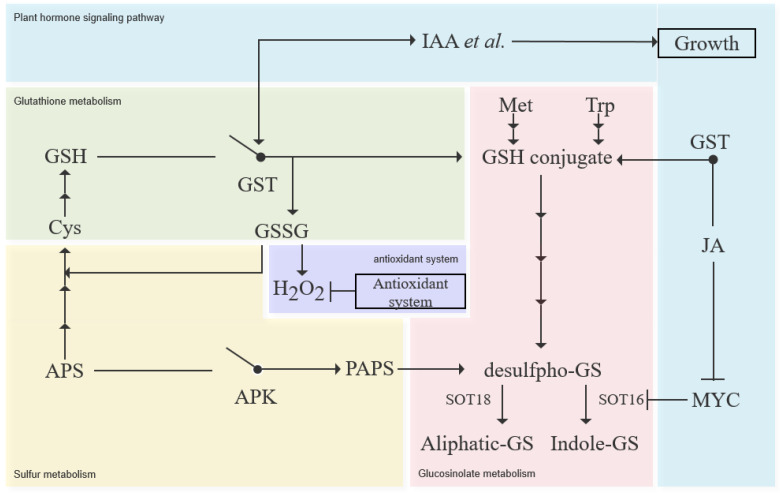

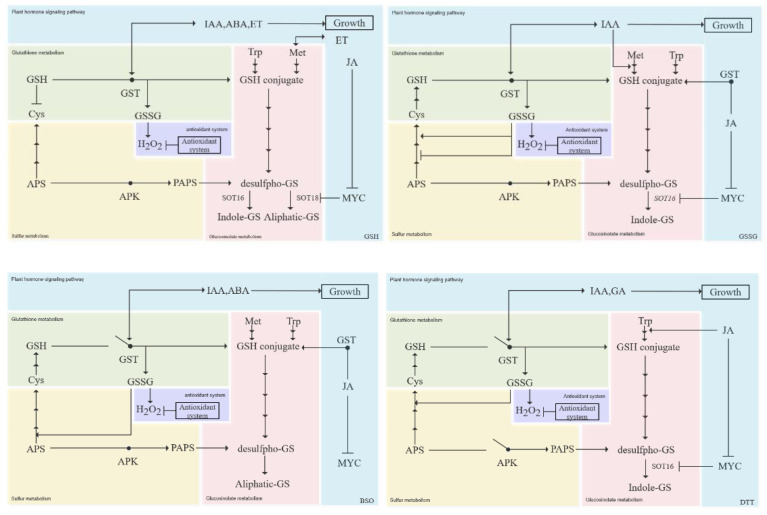
Model of how GSH keeps balance between antioxidant response and GS metabolism in pakchoi.

It is known that plant growth and development of GST is induced by plant hormones, such as IAA, CK, SA, MeJA, ET and other hormones, indicating that plant GST may play a dynamic role in plant growth and development [59]. GST is also involved in secondary metabolism and signal transduction. For example, GST can rapidly combine with ITC to form complex (ITC-GSH), driving them to be passively absorbed into cells [60]. Mueller et al. found that *AtGSTF8* can catalyze the coupling of GSH with two stress signaling molecules [61]. GST is also involved in plant antioxidation. *gmgstu10-10* in soybean can effectively catalyze under reduced GSH enrichment (e.g., oxidative stress) [62]. Combined with the model presented in Figure 12 results above, it is speculated that GST is the fulcrum of GSH regulating the balance between oxidative stress and GS metabolism.

## 5. Conclusions

In conclusion, the following results can be summarized: (1) GSH can improve the oxidative stress resistance of plants as priming; (2) in case of abiotic stress, GSH will preferentially activate the resistance system and reduce the activity of secondary metabolism through the GST protein family, so as to balance the total material consumption; (3) GSH inhibits GS synthesis through the GST and APK family.Due to these results, we propose the model in Figure 12. GSH will first tend to regulate the gene expression of the plant resistance pathway, especially antioxidant pathway, and inhibit the key steps of GS synthesis through the GST protein family, so as to obtain the dynamic balance between the resistance pathway and metabolic pathway.

## Figures and Tables

**Table 1:** Antioxidant-related gene expressions; values are presented as L2FC relative to CK (0 h).

Gene Name	Treatment	log2FoldChange (Relative to 0 h)				
		1 h	3 h	6 h	12 h	24 h
*CXXS1*	H_2_O	1.245	−0.41	2.155	0.728	0.227
	GSH	0.467	2.380	2.180	1.362	0.158
	GSSG	1.191	1.995	2.249	1.156	0.463
	BSO	0.963	1.127	2.733	1.031	0.620
	DTT	1.576	2.301	2.503	1.086	0.605
*CAT1*	H_2_O	0.702	0.599	0.933	−1.635	−0.912
	GSH	−0.785	0.975	1.027	−0.259	1.228
	GSSG	0.237	0.654	0.052	−0.191	1.645
	BSO	0.096	0.338	1.448	−0.016	1.845
	DTT	0.584	0.492	1.027	0.828	1.759
*RbohD*	H_2_O	3.632	2.442	2.230	−0.81	1.640
	GSH	3.018	1.736	2.226	−1.32	1.983
	GSSG	2.873	2.918	0.231	−0.35	1.580
	BSO	3.705	3.304	2.198	0.095	2.692
	DTT	2.908	2.530	2.508	0.852	2.665
*CSD1*	H_2_O	−0.94	−0.85	−1.99	−0.61	0.324
	GSH	−0.71	1.162	0.659	−1.31	1.667
	GSSG	0.548	−1.68	1.047	0.494	0.631
	BSO	−0.81	−1.26	−1.54	0.791	−0.29
	DTT	0.897	−0.35	−1.38	−2.27	0.990
*FSD3*	H_2_O	−1.60	−0.05	1.351	1.671	−0.36
	GSH	−0.63	−1.68	1.191	1.200	0.156
	GSSG	0.112	−0.98	2.273	2.016	1.559
	BSO	−0.12	0.378	0.689	1.849	−0.58
	DTT	0.513	0.061	0.903	0.040	1.526
*GPX6*	H_2_O	1.051	1.291	1.559	−0.28	0.562
	GSH	0.895	1.430	1.589	−0.97	1.112
	GSSG	0.753	1.916	0.802	−0.51	0.963
	BSO	0.774	1.500	1.654	−0.62	1.873
	DTT	0.890	1.430	1.269	0.336	1.779
*DHAR1*	H_2_O	−0.24	−0.11	0.297	0.321	2.152
	GSH	−0.06	0.583	0.603	−0.12	1.874
	GSSG	−0.39	0.149	0.050	−0.40	1.711
	BSO	0.095	−0.01	0.276	−0.79	2.278
	DTT	0.890	1.430	1.269	0.336	1.779
*GR*	H_2_O	−0.30	0.344	0.832	0.437	0.032
	GSH	0.467	1.721	1.202	0.515	0.669
	GSSG	0.343	1.263	1.620	0.245	−0.10
	BSO	1.018	0.183	1.031	0.093	0.693
	DTT	0.416	0.059	1.214	0.673	0.465
*APX1*	H_2_O	0.167	−0.16	0.995	0.277	1.729
	GSH	0.486	0.460	1.011	0.297	2.586
	GSSG	0.484	0.630	1.441	0.762	2.398
	BSO	0.127	0.636	1.189	0.803	3.094
	DTT	0.661	1.054	2.085	0.689	3.178

## Data Availability

The datasets supporting the results of this article are available at the Sequence Read Archive (SRA) database of National Center for Biotechnology Information (NCBI; https://www.ncbi.nlm.nih.gov/ accessed on 27 May 2022) under project accession number PRJNA855156.

## Supplementary Materials

The following supporting information can be downloaded at: https://www.mdpi.com/article/10.3390/antiox11071322/s1, Figure S1: Kmeans cluster analysis of GSH metabolism, antioxidant system, sulfur metabolism, plant hormone signaling pathway and GS metabolism pathways under GSH(a), GSSG(b), BSO(c) and DTT(d) treatments; Figure S2: Expression of typical DEGs response to spraying treatments at 1, 3, 6, 12 and 24 h; Table S1: The amplification primer of qRT-PCR for Pakchoi; Table S2: The number of up-regulated and down regulated genes at different time points under different treatments; Table S3: DEGs in stress response to spraying treatmentsat 1, 3, 6, 12, and 24 h; Table S4: DEGs of ‘metabolism’ and ‘growth’ response to spraying treatments at 1, 3, 6, 12, and 24 h.

## Abbreviations

GSH	Glutathione
ROS	Reactive oxygen species
GS	Glucosinolate
AsA	Ascorbate
SOD	Superoxide dismutase
CAT	Catalase
APX	Ascorbate peroxidase
DHAR	Dehydroascorbate reductase
GR	Glutathione reductase
GPX	Glutathione peroxidase
GST	Glutathione S-transferase
POD	Peroxidases
GSSG	Glutathione disulfide
JA	Jasmonic acid
BSO	L-Buthionine-sulfoximine
DTT	Dithiothreitol
MDA	Malondialdehyde
H_2_O_2_	Hydrogen peroxide
O_2_^•−^	Superoxide
PCA	Principal component analysis
DEGs	Differentially expressed genes
GO	Gene ontology
SA	Salicylic acid
ET	Ethylene
JA	Jasmonate
ABA	Abscisic acid
IAA	Indole-3-acetic acid
